# Splenic marginal zone lymphoma: a case report and literature review

**DOI:** 10.1186/s12957-020-02030-3

**Published:** 2020-10-01

**Authors:** Shiyu Zhang, Zefeng Xuan, Liang Zhang, Jiahua Lu, Penghong Song, Shusen Zheng

**Affiliations:** 1 The First Affiliated Hospital, Zhejiang University School of Medicine, Division of Hepatobiliary and Pancreatic Surgery, Department of Surgery, Zhejiang Province, China; 2 NHC Key Laboratory of Combined Multi-organ Transplantation, Zhejiang Province, China; 3 Key Laboratory of the diagnosis and treatment of organ Transplantation, Research Unit of Collaborative Diagnosis and Treatment For Hepatobiliary and Pancreatic Cancer, Chinese Academy of Medical Sciences, Beijing Province, China; 4 Key Laboratory of Organ Transplantation, Research Center for Diagnosis and Treatment of Hepatobiliary Diseases, Zhejiang Province, 310003 Hangzhou China

**Keywords:** Splenic marginal zone lymphoma, Management, Staging, Case report

## Abstract

**Background:**

Splenic marginal zone lymphoma (SMZL) is a rare non-Hodgkin lymphoma, and much little is known about its clinical characteristics and management strategies. Here we present a case of SMZL and review relevant literature to provide a better recognition of this disease entity.

**Case presentation:**

A 49-year-old Chinese female was admitted to our hospital with complaints of abdominal distension and acid reflux. Physical examinations and imaging investigations suggested the presence of splenomegaly. Laboratory workups revealed mildly reduced white blood cell count otherwise was not remarkable. The patient underwent splenectomy. Histological examination combined with immunohistochemical analysis of the resected spleen confirmed the diagnosis of SMZL. The patient recovered uneventfully during follow-ups.

**Conclusions:**

Due to the rarity and unspecific clinical features, SMZL is extremely challenging to be diagnosed preoperatively. Patients with SMZL are generally associated with favorable prognosis. Combining the staging characteristics of non-Hodgkin’s lymphoma and splenic primary lymphoma may assist in clinical staging management of SMZL.

## Background

Lymphoma is a malignant tumor originating from the lymphatic hematopoietic system. According to the different cell sources, it is divided into non-Hodgkin lymphoma (NHL) and Hodgkin lymphoma (HL). Primary splenic lymphoma is a rare type of malignant lymphoma, which involves only the spleen and splenic hilar lymph nodes. However, splenic marginal zone lymphoma (SMZL) is even rarer.

SMZL represents a rare chronic B lymphocyte proliferative disease, which only accounts for about 1–2% of non-Hodgkin’s lymphoma [1]. The mean age of SMZL onset is about 65 years. There was no significant difference between men and women. About 25% of patients with early SMZL have no significant special discomfort; some people might only show the upper abdominal discomfort, such as abdominal pain and distention; and other patients may be accompanied by splenomegaly, emaciation, fatigue, weight loss, or other manifestations.

The cell origin of SMZL remains not fully clear; some scholars believe that it originates from memory B lymphocytes in the marginal zone of secondary lymphoid follicles [2]. The pathogenesis of the disease is still in the process of exploration. Many studies believe that its pathogenesis may be closely related to 7q distortion [3]. Although it is an indolent lymphoma, about 10% of patients can be converted to diffuse large B cell lymphoma [4], and if the patient has B symptoms, it will lead to the deterioration of the condition. Overall, the prognosis of SMZL is better than other types of lymphoma. If the patients show no obvious hypersplenism or disease progression, the median survival time of the disease may be more than 10 years [5, 6].

Due to the rarity of this disease entity, little was known about its diagnosis and treatment. At present, the treatment of SMZL is relatively limited. If the patient has no obvious hypersplenism or disease progression, long-term follow-up is recommended firstly. General treatments include surgical treatment, radiation therapy, and systemic chemotherapy. For most patients, surgery is an effective way to both diagnosis and treatment of SMZL, and postoperative adjuvant chemotherapy can effectively prolong the survival rate.

At present, the current staging system for the SMZL is completely exploiting from the clinical experience of NHL. Due to the involvement of the spleen, SMZL was clearly classified as stage III NHL according to the staging system for the NHL. It is well recognized that patients with stage III NHL were associated with a dismal prognosis, with a 5-year survival of only 50%. By stark contrast, the SMZL was fairly described as a relatively biologically indolent tumor, despite the nature of NHL, and usually portended a favorable prognosis. As such, the implementation of the staging system of NHL for SMZL may seem not reasonable. Given this background, we herein reported a case of SMZL and meanwhile reviewed the existing literature as well as our center experiences to propose a clinical staging system of SMZL, which may potentially assist in the management of this uncommon entity.

## Case presentation

**Fig. 1:**
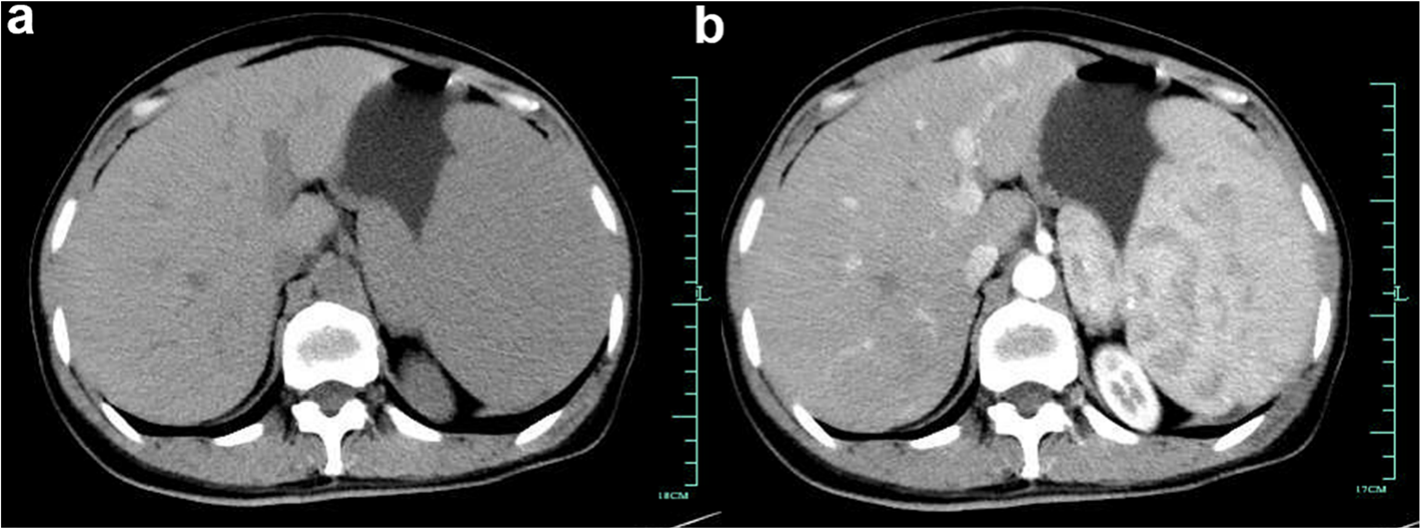
Abdominal enhanced computed tomography (CT) images. The images showed the presence of splenomegaly (**a**), without significant enhancement (**b**)

A 49-year-old Chinese female was presented to a local hospital with complaints of occasional abdominal distension and acid reflux of 1-month duration. The patient had no weight loss, anemia, or fever and denied any family history of cancer. The medical history of the patient was negative. Ultrasonography (US) and computed tomography (CT) revealed splenomegaly. A tentative diagnosis of blood system diseases was made, and then the patient underwent a bone marrow biopsy, which suggested that granulocytes were normal in proportion, and no obvious atypical cells were found. Although repeated biopsy was suggested, the patient refused and was referred to our hospital. On admission, physical examination revealed that the abdomen of the patient was soft and the spleen can be touched under the left costal arch. Laboratory tests showed white blood cell count 3 × 10^9^/L, hemoglobin (HB) 125 g/L, and platelet count 151 × 10^9^/L. The liver function and coagulation function were within normal limits. US indicated that the spleen was 5.7 cm in thickness, with smooth contour, uniform, and meticulous echo of the spleen parenchyma. Enhanced CT (Fig. 1) was performed and suggested splenomegaly (maximum diameter 23 cm), without swollen lymph nodes in the abdomen.

**Fig. 2:**
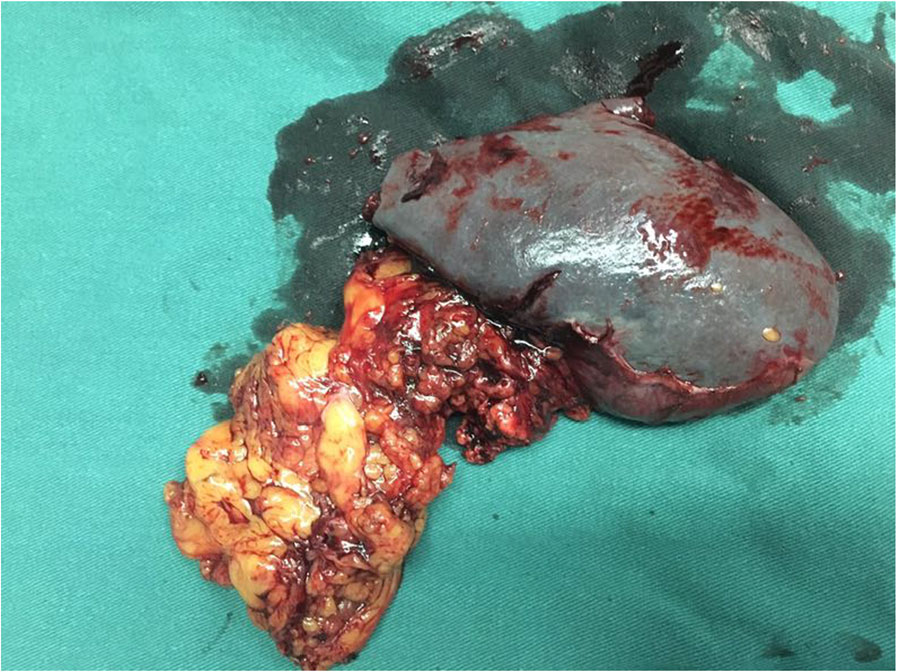
Macroscopic examination of the postoperative specimen. The resected spleen measured 22 × 13 × 5 cm in size

**Fig. 3:**
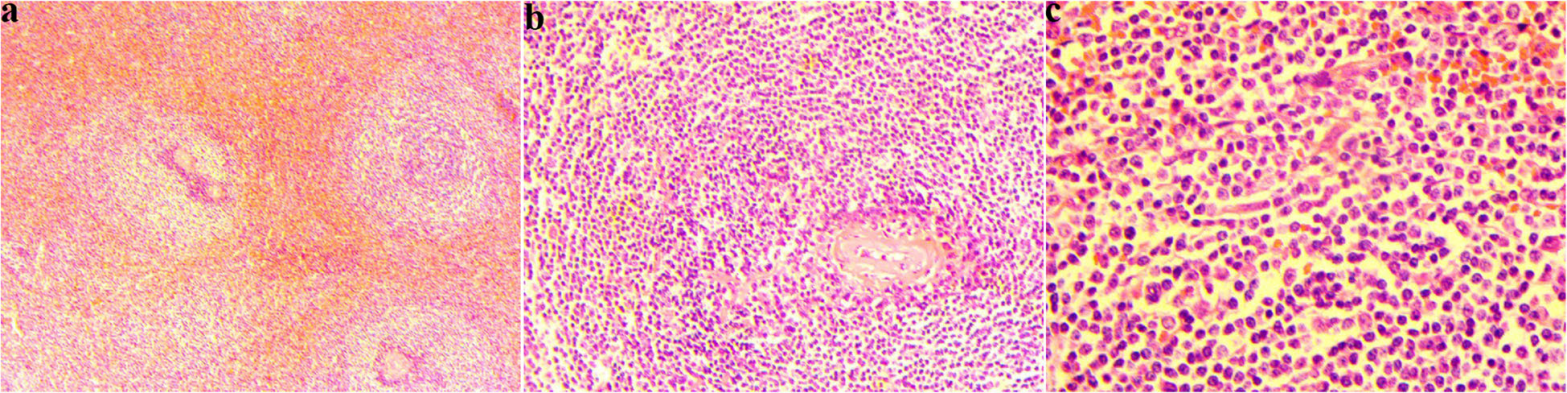
Histopathological analysis of the surgical specimen. The tumor showed nodular growth pattern (hematoxylin and eosin (H&E), × 40) (**a**). The white pulp of the spleen was significantly enlarged, where a large number of infiltrating tumor cells were observed. And the tumor cells were small and round in size (H&E; **b** × 100, **c** × 200)

**Fig. 4:**
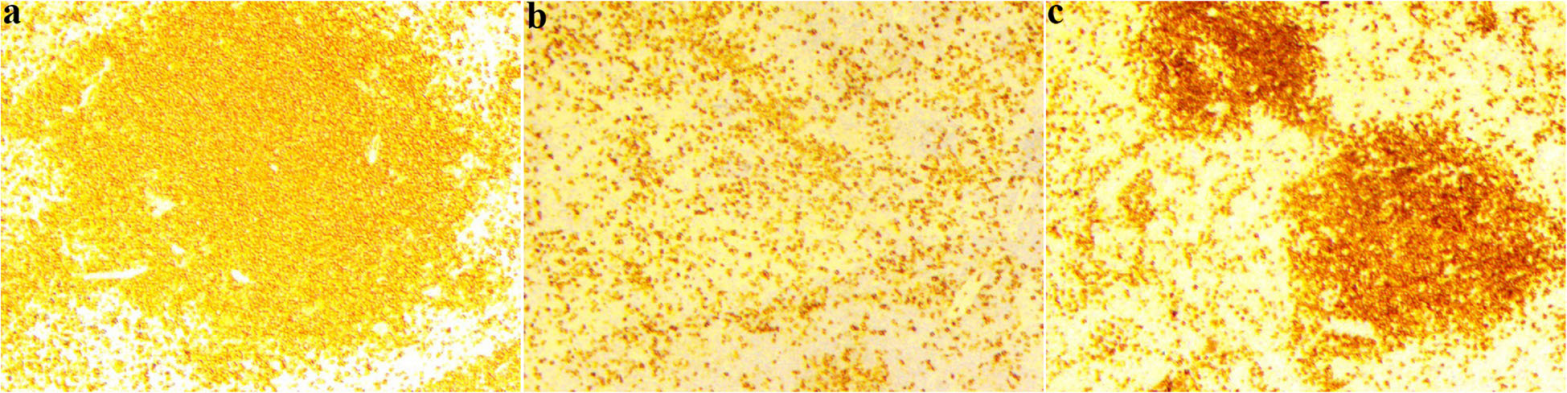
Immunohistochemical examinations of the surgical specimen. Immunohistochemical staining revealed that tumor cells were strongly positive for CD20 (**a**), CD43 (**b**), and CD79a (**c**) (magnification, × 100)

In order to make a definite diagnosis, the patient underwent splenectomy. During the operation, we found that the spleen is obviously enlarged, and the specimen of the resected spleen was roughly dark reddish (Fig. 2). The final pathological examination showed that both number and size of the splenic white pulp were increased, and small lymphocytes proliferate in the nodules (Fig. 3). The involvement of the splenic hilar lymph nodes was also noted. Immunohistochemistry was positive for Ki67, Bcl-2, CD43, CD79a, and CD20 (Fig. 4). Eventually, the patient was diagnosed with SMZL.

The postoperative courses were uneventful. The patient’s white blood cell count increased to 8.1 × 10^9^/L, and the platelet count increased to 465 × 10^9^/L. During 12 months of follow-ups, the patient showed no signs of recurrence or spread.

## Discussion and conclusions

The spleen represents the largest solid immune organ of the body. It contains numerous lymphocytes and macrophages and serves as the center of cellular immunity and humoral immunity [7, 8]. Located between the white pulp and the red pulp of the spleen, the marginal region is approximately 100 μm in width and is the main anatomical channel for the peripheral lymphocytes to enter the lymphatic system of the spleen. Functionally, the marginal region of the spleen provides a platform for the recognition of antigens and activation of immune reactions [9].

Due to the rarity of SMZL, much little was known about its pathogenesis. NOTCH gene mutation [10, 11] and the deletion of 7q31-7q32 [12] were previously suggested to be involved in the development and progression of SMZL. In addition, patients with SMZL were frequently diagnosed as co-infected with hepatitis C virus (HCV) [13, 14]. Nonetheless, these data should be interpreted cautiously because of the limited numbers of study subjects. In this case, the patient did not have HBV/HCV infection, and the previous history was not remarkable.

Splenomegaly is the most typical symptom of SMZL, and some patients present with decreased appetite, fever, night sweats, and sudden weight loss. US is widely used in clinical practice, and the performance of SMZL is usually low or anechoic [15]. CT mainly shows splenomegaly, decreased lesion density on plain scan, and mild to moderate enhancement on enhanced scan [16]. PET/CT shows increased splenic uptake of ^18^F-FDG, which is higher than the liver. Indeed, Rini et al. reported that PET/CT had a 100% sensitivity to lymphoma of the spleen, compared with that of 57% for conventional CT scans [17]. However, because of the high price and radiation of PET/CT, it is not routinely recommended as the first choice in the clinical practice [15, 18]. Due to unspecific imaging features, diagnostic splenectomy is still the preferred diagnostic method. Splenic histology is characterized by small lymphocyte infiltration with nodular hyperplasia. It is also suggested to use spleen biopsy to preoperatively diagnose SMZL regarding the advantages of reducing the risks of post-splenectomy complications typical of intraperitoneal hemorrhage and infection [19].

Due to the rarity of SMZL and its unspecific clinical manifestation, until now, there is still no uniform diagnostic standard. In 2008, Matutes et al. proposed the minimum diagnostic criteria for SMZL [17], which were based on spleen biopsy results and CLL immunophenotype score. However, the clinical classification of SMZL has yet been inconsistent. SMZL is a type of non-Hodgkin lymphoma (NHL) and therefore should be staged according to NHL criteria. According to the NHL criteria (staging system), when the tumor cells invade the spleen, its staging would be classified as at least stage III NHL; stage III is usually associated with a dismal prognosis and the 5-year survival time is only about 50%. However, SMZL is a relatively indolent lymphoma and had a favorable prognosis. As such, it may seem inappropriate to apply NHL standards for staging SMZL. On the other hand, SMZL is also a type of primary splenic lymphoma, and therefore, its clinical staging complies to Ahmann’s staging criteria, which is widely used to primary splenic lymphoma irrespective of NHL. Therefore, we improved Ahmann’s staging by taking different sites of lymphoma into consideration. But this is just our initial idea and still needs further confirmation. In our own staging standard (see Table 1 for details), SMZL is divided into 3 phases, according to different parts of tumor cell invasion. When lymphoma is limited to the spleen (stage I), the patients may have a favorable prognosis.

**Table 1:** The staging criteria for SMZL

Stage І	Tumors were confined to the spleen only; no metastatic lesions were found in the splenic lymph nodes and outside the spleen.
Stage ІІ	The tumor involved in the splenic lymph nodes but no other parts of the lymph nodes or the external organs of the lymphatic system.
Stage III	Lymph node involvement outside the spleen or extra-lymphatic organs and their associated lymph nodes (liver and bone marrow).

If the patient has fever, night sweats, and clinical manifestations of weight loss in the past 3 months, it is classified as class B; otherwise, it is class A

**Fig. 5:**
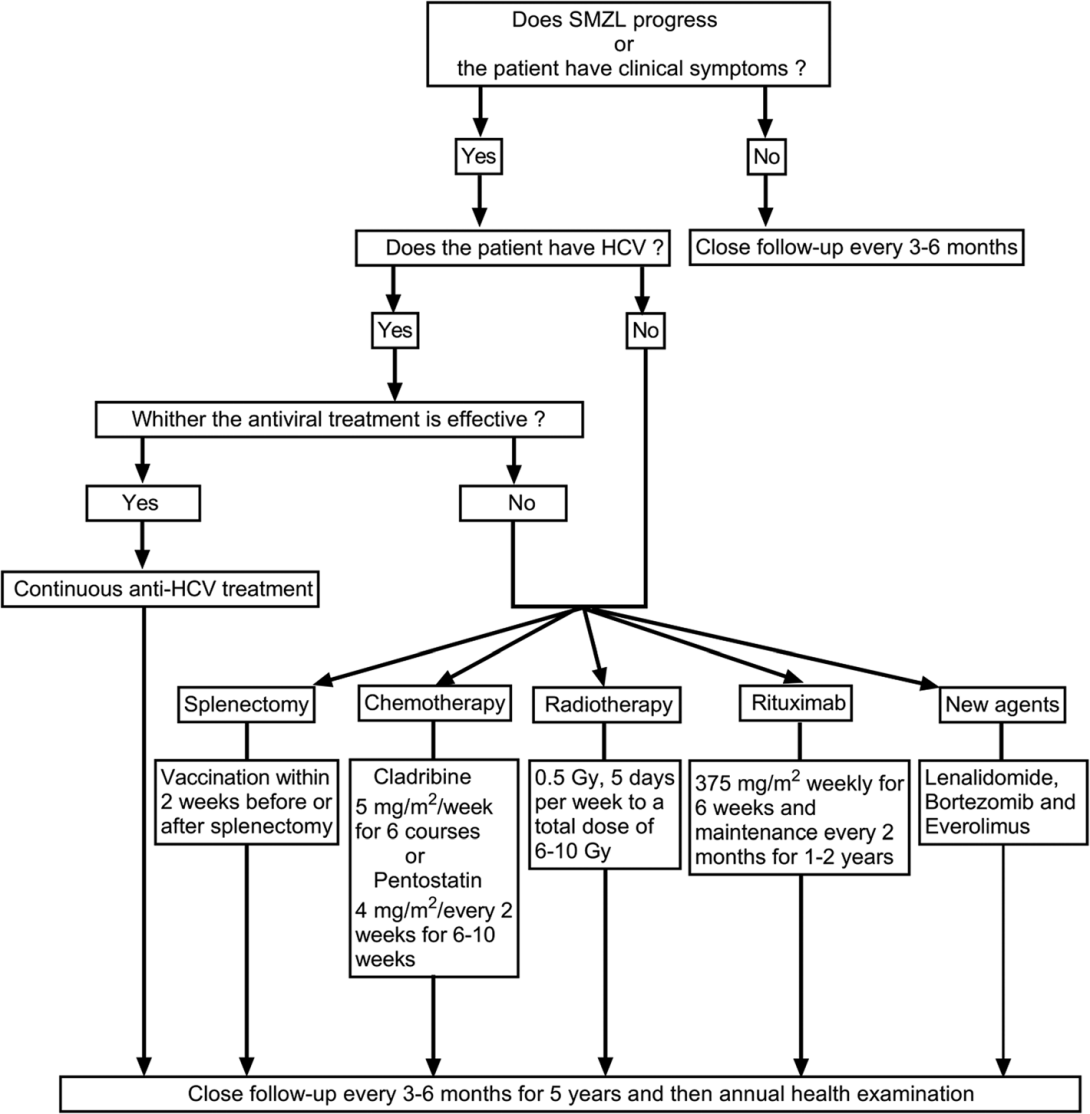
Flow diagram depicting the management of patients with SMZL. A brief summary of the management strategy for the SMZL

Given the paucity of SMZL, no consensus of treatments can be reached. For asymptomatic patients, conservative treatment is recommended and close follow-up should be conducted about every 3–6 months. The follow-up examinations should include ultrasound, blood routine, and so on [20]. If a progressive change in the patient’s condition is found during follow-up (such as hypersplenism and B symptoms), then hospitalization will be recommended [21]. For HCV-positive SMZL patients with no obvious surgical indications (such as hypersplenism), interferon alone or combined with ribavirin for anti-HCV therapy may be considered as an effective option [22]. Antiviral therapy could achieve the clearance of serum HCV RNA and, at the same time, contribute to the remission of SMZL. Rossotti et al. reported that after 16 weeks of antiviral therapy for SMZL patients with coexistent HCV infection, the serum viral load of HCV in these patients became undetectable and the size of the spleen normalized [23]. For HCV-negative SMZL patients who have obvious splenomegaly, splenectomy is still the first choice for treatment. It has been reported that splenectomy could significantly prolong the patient’s survival, especially for those with preoperative thrombocytopenia [24]. Splenectomy could achieve the purposes of getting a definitive diagnosis, reducing the tumor burden and relieving thrombocytopenia-associated symptoms. Additionally, SMZL patients who received splenectomy as the first-line therapy were reported to have a median survival time of 8.25 years, with a 5-year overall survival rate of 84% [25]. In another study, Olszewski et al. reported that the disease-free survival for SMZL patients undergoing splenectomy generally exceeded 5 years [26]. However, it should be noted that severe infection after splenectomy, also known as overwhelming post-splenectomy infection (OPSI), is highly lethal, with an estimated mortality rate of 5% [27, 28]. The majority of OPSI is caused by encapsulated bacteria, typically of the pneumococcus, meningococcal C pathogens and hemophilus influenza B [29]. To prevent the development of OPSI, vaccination against encapsulated bacteria is mandatory within 2 weeks before or after splenectomy and then once a year for 5 consecutive years [29]. While for patients who cannot tolerate surgery or experience tumor recurrence after surgery, chemotherapy should be considered. A retrospective study by Riccioni et al. reported that following cladribine treatment (5 mg/m^2^/week, 6 courses), the partial remission rate and complete remission (CR) rate of SMZL patients could achieve 60% and 20%, respectively [30]. Iannitto et al. used pentostatin (4 mg/m^2^/every 2 weeks) to treat SMZL for 6 to 10 weeks, and the overall remission rate (ORR) was about 68% [31]. Recently, with the advent of the anti-CD20 monoclonal antibody (mainly rituximab), the paradigm of systemic therapy for SMZL has changed, and rituximab-based therapy has become an effective alternative to splenectomy [32]. The standard rituximab monotherapy should include induction and maintenance phases. Rituximab is given at the standard dose (375 mg/m^2^/week) once per week for 6 weeks during the induction phase. Then, in the maintenance phase, rituximab is given at the standard dose (once every 2 months) for 1–2 years [33, 34]. Kalpadakis et al. reported that treatment with rituximab monotherapy could result in a 5-year overall survival rate of 92% [33]. In addition, for SMZL patients with constitutional symptoms, rituximab combined with chemotherapy may be considered. Brown et al. reported that after combined treatment strategy (fludarabine combined with rituximab), the ORR was up to 85% [35]. Furthermore, splenic irradiation has been suggested as an effective treatment option for SMZL patients who respond poorly to the systemic therapy. Oliveira et al. reported that splenic radiotherapy (1.5 Gy, 5 days a week for 3 weeks) could effectively diminish the volume of the enlarged spleen [36]. It has also been reported that the low-dose spleen radiotherapy regimen (0.5 Gy, 5 days per week to a total dose of 6–10 Gy) could provide effective palliation for SMZL without excessive toxicity [37, 38]. And, more remarkable, repeated irradiation seems to be safe for those patients with no durable response [38]. More recently, novel agents for SMZL have been designed and tested in several clinical studies, including lenalidomide, bortezomib, and everolimus [32]. Although these studies are just in infancy, the results seem promising. In Fig. 5, we summarize the specific treatment for SMZL.

In order to know more about SMZL, we collected information of 16 patients who failed to be diagnosed before operation from the First Affiliated Hospital of Zhejiang University since 2010. Most patients were between 50 and 70 years old, which was consistent with literature reports. Patients with SMZL presented with abdominal discomfort, fever, and other non-specific manifestations (Table 2). Almost all patients presented with splenomegaly, and the largest case even reached 40 × 20 × 15 cm (Table 3). In terms of immunohistochemistry, almost all tumor tissues showed positive for CD20 and CD79a (Table 4).

**Table 2:** Summary of the demographic characteristics of patients with SMZL in our center

Variables	Number of cases	Percentage
Gender		
Male	4	25.0
Female	12	75.0
Age		
0–49	4	25.0
50–70	11	68.75
> 70	1	6.25
Main symptoms		
No obvious symptoms	3	18.75
Abdominal discomfort	4	25.0
Fever	2	12.5
Weight loss	2	12.5
Changes in blood routine results	3	18.75
Fatigue	2	12.5

**Table 3:** Summary of the size of the surgically resected spleen for patients with SMZL

Spleen size	Centimeters
Min	18 × 13 × 5
Max	40 × 20 × 15
Average	24.9 × 15.8 × 8.6

**Table 4:** The immunohistochemical staining results of SMZL

Immunohistochemistry	Proportion
CD20 (+)	16/16 (100%)
CD79a (+)	11/16 (68.75%)
CD43 (+)	9/16 (56.25%)

To sum up, SMZL is a low-grade malignant non-Hodgkin lymphoma. Due to lack of typical clinical manifestations, it is necessary to combine US, CT, PET/CT, and other imaging examinations, laboratory examinations, bone marrow biopsy, immunohistochemistry, histopathology, and other comprehensive examinations for the diagnosis of SMZL. We merged the staging of non-Hodgkin’s lymphoma and primary splenic lymphoma and proposed a staging for SMZL, but further confirmation is needed in the future.

## Data Availability

Not applicable.

## Abbreviations

CT: Computed tomography
CR: Complete remission
HB: Hemoglobin
HCV: Hepatitis C virus
HL: Hodgkin lymphoma
NHL: Non-Hodgkin lymphoma
OPSI: Overwhelming post-splenectomy infection
ORR: Objective response rate
PET: Positron emission tomography
SMZL: Splenic marginal zone lymphoma
US: Ultrasonography
